# Single molecule sequencing-guided scaffolding and correction of draft assemblies

**DOI:** 10.1186/s12864-017-4271-8

**Published:** 2017-12-06

**Authors:** Shenglong Zhu, Danny Z. Chen, Scott J. Emrich

**Affiliations:** 1 Department of Computer Science & Engineering, University of Notre Dame, 244 Fitzpatrick Hall, Notre Dame, 46556 IN USA; 2 Department of Computer Science & Engineering, University of Notre Dame, 326E Cushing Hall, Notre Dame, 46556 IN USA; 3 Department of Computer Science & Engineering, University of Notre Dame, 211B Cushing Hall, Notre Dame, 46556 IN USA

**Keywords:** Genome improvement, Single molecule sequencing, Sequence assembly, Genome scaffolding

## Abstract

**Background:**

Although single molecule sequencing is still improving, the lengths of the generated sequences are inevitably an advantage in genome assembly. Prior work that utilizes long reads to conduct genome assembly has mostly focused on correcting sequencing errors and improving contiguity of *de novo* assemblies.

**Results:**

We propose a disassembling-reassembling approach for both correcting structural errors in the draft assembly and scaffolding a target assembly based on error-corrected single molecule sequences. To achieve this goal, we formulate a maximum alternating path cover problem. We prove that this problem is *NP*-hard, and solve it by a 2-approximation algorithm.

**Conclusions:**

Our experimental results show that our approach can improve the structural correctness of target assemblies in the cost of some contiguity, even with smaller amounts of long reads. In addition, our reassembling process can also serve as a competitive scaffolder relative to well-established assembly benchmarks.

## Background

Paired end sequencing, where shorter, high quality sequences are generated from both ends of a longer DNA fragment, has been used to improve genome assemblies (see [1] for a review). This method was a significant advance since it allowed scaffolding [2] and improved assembly quality assessment [3]. It also helped resolve many repetitive sequences that are smaller than the DNA fragments provided to sequencing [1].

Although the total cost of sequencing has decreased substantially, the DNA fragment sizes suitable for inexpensive sequencing have remained similar. As a result, repetitive sequences larger than a few thousand bases in extant genomes largely remain either incorrect or absent. Prior work has focused on combining different assemblers or picking the best assembly (see [4] for a review), but these approaches cannot fix errors (or omissions) produced by all assemblers [2].

Newer sequencing methods, such as Illumina’s long reads (e.g., [5]) or the more recent 10X Genomics Chromium platform, will indirectly increase fragment sizes through localized assembly. If repeats are spread throughout the genome, instances of repeats should be unique in each subproblem (reconstructed fragment) and therefore can be assembled well. If they are not distributed throughout, as in the case of complex genomes like maize [6], such approaches will suffer from the same pitfalls of traditional assembly.

In this paper, we present an alternative framework that uses single molecule sequencing (SMS) data [7–10]. In contrast to paired end sequencing, SMS platforms like Oxford Nanopore generate low quality sequences that currently can be as large as one hundred thousand nucleotides. Because the sequences are generated from actual DNA molecules, they are ideal to resolve local tandem repeats, transposon structure(s), and small-scale inversions and translocations. Most importantly, this new method can in theory be applied to any extant genome to improve quality. We demonstrate this on both bacterial and yeast genomes using currently available SMS data.

**Fig. 1:**
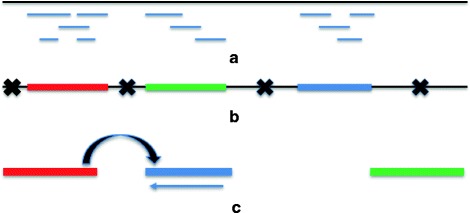
A visual overview. **a** Alignment of long reads to the target assembly. **b** Extraction of structurally consistent segments from the target assembly. **c** The reassembly of the consistent portions has two scaffolds: The first scaffold is formed by the red (1st) segment followed by the reverse complement (indicated by a leftward arrow) of the blue (3rd) segment in **b**, and the gap induced by disassembling is then filled by one or more connecting long reads; the second scaffold is the single green (2nd) segment

To the best of our knowledge, the framework of BIGMAC [11] is the most similar to ours. BIGMAC, however, is designed to improve genome accuracy for metagenomic assembly by extensively relying on the assembly itself. As a result, it can hardly correct structural errors depicted in Fig. 1, and this was validated in a comparison. Individual components of this method are also related to prior work in assembly improvement. For example, our scaffolding step is similar to the likelihood-based gap closing approach of GMcloser [12], similar to [13], as well as the recently reported ScaffMatch [14]. In contrast, we compute overlaps between SMS reads in order to more accurately weight the connections between genome regions. To further validate and compare with prior methods, we also test our approach on a well-established genome assembly benchmark (GAGE [15]) at varied coverages of SMS scaffolding data.

## Results

We evaluate the performance of our program, denoted as SMSC, on both synthetic data and real data as target assemblies. In the case that the experimental results of different program settings are similar, we present only one of them.

### Synthetic data

*Escherichia coli* is a well studied organism with a highly curated genome assembly. For this reason, it is a common benchmark for assembly software. The input to our program is a target assembly with induced structural errors (see below) and Nanopore long reads that have already been corrected by Nanocorr [16].

**Fig. 2:**
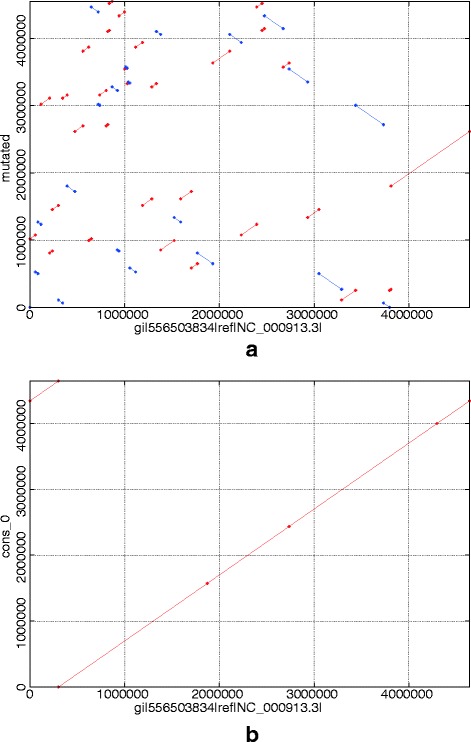
Comparisons generated by Nucmer and delta-filter (with -r and -q options). **a** Comparison of the mutated *E. coli* genome to reference. **b** Comparison after applying our method

To randomly mutate the complete genome of *E. coli* K-12 (accession NC_000913), we wrote a Python script to cut the genome randomly *N*−1 times (*N*=50 in Fig. 2 a). Specifically, if the total length of the remaining genome is *L*, and there are *m* cuts to go, then we chose a cutting point uniformly randomly between length *l* and *L*/(*m*+1) (*l*=10000 when *N*≤400 and *l*=5000 when *N*=500 in Table 1. The reason we chose different *l* is to more accurately represent expected fragmentation post-assembly given the length of the *E. coli* sequence). After this cut, there are (*m*−1) cuts to go. After all the cuts, we then shuffle the pieces and randomly reverse complement some to eliminate known order and orientation. Further, we introduced 2% insertion, deletion, and mismatches each into the new target assembly for correction.

**Table 1:** Correction of mutations of *E. coli* K-12 with two additional downsamples of the 50X data

Coverage	# of mutations	50	100	150	200	300	400	500
50x	scaffolds	1	1	3	1	2	9	7
	Total running time	23m35s	25m	26m37s	27m41s	32m15s	37m51s	38m29s
	Stage 1 time	20m31s	20m4s	19m24s	18m37s	18m25s	17m58s	17m30s
	Stage 2 time	3m4s	4m56s	7m13s	9m4s	13m50s	19m53s	20m59s
	Relocations	1	1	3	2	3	6	5
	Inversions	0	0	0	0	0	0	1
	N50	4641851	4642418	3799940	4641735	4495031	1610858	2378162
25x	scaffolds	3	3	7	16	14	23	39
	Total running time	17m47s	18m47s	20m8s	20m56s	23m31s	27m2s	26m7s
	Stage 1 time	15m52s	15m27s	15m26s	14m50s	14m21s	14m37s	13m13s
	Stage 2 time	1m56s	3m20s	4m42s	6m6s	9m10s	12m25s	12m54s
	Relocations	2	1	2	1	3	7	12
	Inversions	0	0	0	0	1	3	7
	N50	3046572	4641477	4638607	4641947	2781405	906635	1057202
12.5x	scaffolds	20	11	18	31	59	74	120
	Total running time	16m28s	16m43s	17m17s	17m32s	18m32s	19m34s	18m33s
	Stage 1 time	14m31s	13m51s	13m12s	12m37s	11m41s	11m1s	9m45s
	Stage 2 time	1m57s	2m52s	4m5s	4m55s	6m51s	8m33s	8m48s
	Relocations	3	1	3	6	7	9	11
	Inversions	0	0	2	2	1	6	8
	N50	1592081	1150026	719436	730221	385833	276032	118970

The coverage of these Nanopore long reads is 50x, the average length is 4070 base pairs (bp), and the upper quartile is 6231 bp. Figure 2 is a before and after example comparison using these data in our method visualized using mummerplot [17]. Bacterial genomes are circular, so the two genome alignment segments actually represent a single genome. This figure shows that our method disassembled the erroneous genome and then correctly reassembled the consistent segments.

To test the performance of our method with higher fragmentation, we gradually increased the number of mutations in the mutated genome (see Table 1). The numbers of relocations and inversions post correction were assessed by dnadiff, which is another tool in the MUMmer toolkit. Based on the definition of comparisons in [17], translocations are not reported given that *E. coli* K-12 is a single sequence. Because dnadiff does not natively distinguish whether a genome is circular, there will be a relocation if the starting location of our output is different from that of the ground truth. We also noticed that many inferred relocations and inversions are small and localized. These appear to be insertions present in our assembly resulting from either imperfect read alignments or uncorrected errors in the long reads.

To see how the coverage of the long reads affects our method, we downsampled the long reads to 25x and 12.5x, where the 12.5x sampling is a subset of the 25x sample (see Table 1). Because the results of using Nucmer and BLASR as alignment tools are similar for this genome, the results in Table 1 were all produced using Nucmer as the alignment tool.

The results of our experiments show that, as expected, our results depend somewhat on both the coverage of the long read data and the fragmentation/errors present in the target assembly. Higher fragmentation produces shorter fragments, which will lead to shorter validated segments because our conservative method requires 99% of the long reads to align. We expect this could be alleviated somewhat by changing the parameters to be 99% of the smaller of the two sequences or relaxing this threshold.

### Real data

#### *S. cerevisiae* W303

We downloaded the yeast (*S. cerevisiae* W303) draft assembly from [18], and error-corrected PacBio long reads (10x, upper quartile length of 932bp, already corrected by the Celera Assembler PBcR pipeline) from [19]. After running our program on this draft assembly with the error-corrected long reads, the number of contigs increases from 30 in the draft assembly to 294 (using BLASR as alignment tool) and 278 (using Nucmer as alignment tool). Given the short and overall low coverage post long read correction, however, this added fragmentation is consistent with our earlier simulations.

To our best knowledge, there are only draft assemblies available for *S. cerevisiae* W303 [16, 19, 20]. We therefore used its closely related strain *S. cerevisiae* S288 (available at [16]) as in [16]. We then used Quast [21] as the quality assessment tool to determine the overall quality. As for the required independent Illumina pair-end reads for Quast evaluation, we used the S288 data downloaded from NCBI (SRA accession SRR507778).

In this experiment, we also compared our results with another scaffolder ScaffMatch [14], which requires traditional paired end reads for scaffolding. The pair-end reads were downloaded from [16]. Its coverage is 105.5x. Since ScaffMatch can only do scaffolding, we use our disassembler output, i.e., the validated segments, as the common input for both methods. Because our edge weighting is based on alignments between error-corrected PacBio long reads, it can therefore fill gaps. ScaffMatch, on the other hand, can only estimate the size of the gap and insert an appropriate number of padding characters (usually an ‘N’).

Table 2 shows that even with suboptimal long read data (10X coverage, <1kb length), the post-correction result is better than the original target assembly. When using BLASR as the alignment tool, our method performs the best, followed by ScaffMatch, except that SMSC with BLASR produces an inversion. Note that the higher fragmentation of our method is somewhat expected given that we are using less data (10X vs. >105X).

**Table 2:** Errors determined by Quast on assemblies of *S. Cerevisiae* W303

	Draft	Correction	Correction	Correction
	assembly	(BLASR)	(Nucmer)	(ScaffMatch)
Contigs	30	294	278	204
Misassemblies	108	22	64	26
Relocations	37	7	18	9
Translocations	69	14	46	17
Inversions	2	1	0	0
Misassembled	10 865 048	969 713	3 151 961	1 607 623
contig length				

#### *S. aureus* USA300

Since we did not use the exact strain in the comparison above, we decided to also test a well-characterized GAGE benchmark [15]: *S. aureus* USA300.

Its complete genome, a draft assembly, and pair-end reads were downloaded from [15]. Although there are several assemblies on GAGE, we present only the results from the Allpath-LG assembly since there were no detected structural errors and our method worked best with this assembler. The accession number for the raw PacBio long reads data is SRR2063240, which is then error corrected. After correction by Canu [22], the coverage reduces to 28.63X (from over 293X originally).

Here, we compare with ScaffMatch as before. Since we used Canu to correct the PacBio long reads, we also produced a *de novo* assembly for *S. aureus* USA300 based only on long reads. The experiments on the full dataset (the one with coverage of 293.6x of long reads in Table 3) show that SMSC has better N50 value but more pieces. This is because there are 8 contigs of length ≤300 and 2 of length about 1500.

**Table 3:** Quality comparisons for *S. aureus* USA300 assemblies using downsampled PacBio long reads

Coverage	Tools	Contigs	N50	NG50	AvgIdentity	Reloc	Trans	Inv	Total T	Stg1 T	Stg2 T
293.63x	SMSC	13	2585902	2585902	99.99	3	0	0	6m51s	2m12s	4m39s
	Canu	5	1492711	1492711	99.98	2	0	0	36m26s	N/A	N/A
	ScaffMatch	14	1099804	1099804	99.98	28	2	0	N/A	N/A	1m7s
	*ScaffMatch* ^∗^	31	611114	479066	99.98	16	0	0	N/A	N/A	2m12s
17.54x	SMSC	55	91135	91135	99.84	8	0	1	2m15s	1m5s	1m10s
	Canu	52	89147	84231	99.64	2	0	0	6m8s	N/A	N/A
	ScaffMatch	13	1131851	1131851	99.85	30	0	1	N/A	N/A	1m6s
	*ScaffMatch* ^∗^	106	50567	49351	99.87	2	0	0	N/A	N/A	25s
23.47x	SMSC	18	641333	641333	99.94	3	0	0	2m37s	56s	1m41s
	Canu	27	224570	224570	99.85	2	0	0	7m29s	N/A	N/A
	ScaffMatch	14	1091191	1091191	99.94	29	0	0	N/A	N/A	54s
	*ScaffMatch* ^∗^	70	89077	89077	99.95	3	0	0	N/A	N/A	32s
29.31x	SMSC	12	2569515	2569515	99.95	6	0	0	3m6s	1m4s	2m2s
	Canu	15	426754	426754	99.92	2	0	0	9m54s	N/A	N/A
	ScaffMatch	13	1091146	1091146	99.96	34	0	0	N/A	N/A	54s
	*ScaffMatch* ^∗^	55	149552	149552	99.97	6	0	0	N/A	N/A	37s

The ScaffMatch* means the ScaffMatch that takes error-corrected PacBio long reads as input

We also downsampled the raw PacBio long reads to observe the effects of coverage for this genome. Considering that we downsampled only the raw PacBio long reads but not the PE reads, the output of ScaffMatch will appear to be very good. We also take as input for ScaffMatch the error-corrected PacBio long reads. Since this is not a fair usage of ScaffMatch, we will denote it by *ScaffMatch* ^∗^. Table 3 includes our experimental results from 6 to 10% of the full coverage, and from this table one can see that our approach and ScaffMatch have higher contiguity and identity than *de novo* assemblies. ScaffMatch almost always has a constant contiguity because the PE reads were not downsampled. By taking as input the error-corrected downsampled PacBio long reads, *ScaffMatch* ^∗^ outputs improvement of contigs as the downsample-size increases, as anticipated.

## Discussion

Our results indicate that SMSC is competitive with Canu in terms of contiguity and identity when using lower coverage SMS data. Interestingly, and maybe as expected, scaffolding using both SMSC and ScaffMatch is worse than high-depth SMS *de novo* assembly using Canu.

The higher relocations and misassemblies in ScaffMatch seem to be caused by gaps induced by regions missing in mate-pair data. Since ScaffMatch uses the output of the first stage of SMSC as input, and SMSC implements a simpler scaffolding algorithm than ScaffMatch, we expect that our results could improve if we implemented a more complete algorithm, with the structural consistency competitive with Canu. We leave this implementation as future work.

## Conclusion

Here we presented a new framework that can correct structural assembly errors using single molecule sequencing (SMS) data. Further, we show that long reads when applied to an extant genome can fill scaffold gaps and produce higher overall structural consistency. We expect this framework to be more useful for researchers who have invested heavily in a draft genome and do not want to completely re-sequence this genome.

## Methods

In general, our method consists of disassembling and reassembling genomic regions (Fig. 1). In the first step, we locate structurally consistent segments in an assembly (Fig. 1 a) and extract these regions (Fig. 1 b), thus disassembling the input assembly into smaller, validated portions (or contigs). In the second step, we reassemble the consistent regions (Fig. 1 c) by applying an independently derived path cover model first proposed as a scaffold problem in [14] and fill the gaps. The details of our algorithms are presented below.

### Disassembling

#### Long-read alignment

We first align error-corrected long reads to a target assembly (see Fig. 1 a), using either Nucmer [17] or BLASR [23] (any long-read alignment tool could in theory be used here). The output of Nucmer/BLASR can be viewed as a sequence of aligned blocks (*DL*_*i*_, *DR*_*i*_, *RL*_*i*_, *RR*_*i*_), such that the nucleotides in the range [*DL*_*i*_,*DR*_*i*_] of the target assembly are aligned to the long read in the range [*RL*_*i*_,*RR*_*i*_]. If there are overlaps between consecutive aligned blocks, either in the draft sequence or in the long read, we cut off the left ends of the blocks on the right. We then attempt to close any small gaps between these aligned blocks via the Needleman-Wunsch Algorithm, and call the resulting alignment region an alignment segment.

An alignment segment is kept and used if for any sequence in the target assembly and a long read, the following hold: *i*) For any gap of length *g*_1_ between two aligned blocks [*DL*_*i*_,*DR*_*i*_] and [*DL*_*i*+1_,*DR*_*i*+1_] in the draft assembly, and for the corresponding gap between [*RL*_*i*_,*RR*_*i*_] and [*RL*_*i*+1_,*RR*_*i*+1_] of length *g*_2_ in the long read, the value |*g*_1_−*g*_2_| is within a chosen threshold (30 in our experiments); *ii*) the sum of all alignment blocks for a long read divided by the length of the long read is larger than a chosen threshold (0.99 in our experiments). After picking out these good alignment segments, we claim that if there are two aligned segments overlapping by at least *δ* (also a threshold, 50 in our experiments) in the target assembly, then these two aligned segments in the assembly should be merged.

Since we do not have a quantitative measure for block quality, the choices of these thresholds are based on observations derived from custom visualizations of the alignment results. In general, the first threshold makes sure that if the target assembly is of high quality, then there are not too many pieces after disassembly. The second threshold guarantees that the long reads are well-contained in the target assembly. The third threshold eliminates overlaps that are not trustworthy while trying to retain contiguity. Using error-corrected long reads maximizes the number of obtained alignments and is not a requirement for this step.

The time complexity of the alignment step depends on the tool used (e.g., BLASR or Nucmer). Sorting the aligned blocks takes *O*(*a* log*a*) time, where *a* is the total number of aligned blocks. Cutting off the overlaps takes *O*(*k*) time, where *k* is the number of insertions in all the aligned blocks [*DL*_*i*_,*DR*_*i*_,*RL*_*i*_,*RR*_*i*_]. Closing the gaps takes *O*(*l*_1_ *l*_2_) time for each gap, where *l*_1_=*DL*_*i*+1_−*DR*_*i*_−1 and *l*_2_=*RL*_*i*+1_−*RR*_*i*_−1. Theoretically, in the worst case, the time bound could be *O*(*MN*), where *M* is the total length of the target assembly and *N* is the total length of all the long reads; but this is unlikely to happen in practice because the Needleman-Wunsch Algorithm will not be applied if a contig and a long read are not already aligned by BLASR or Nucmer. Another task is to determine whether an alignment segment should be kept, which takes linear time to handle. In general, this alignment step takes *O*(*MN*) time in the worst-case, and may run faster in practice.

#### Segment extraction

Once we obtain validated segments from the target assembly, we extract these segments and discard the remaining untrustworthy regions (see Fig. 1 b). We support two options for extracting structurally validated segments. The first one is simple extraction. This option is preferred if the quality of the target draft assembly is fairly high. The second option is to generate a new consensus from the long read data aligned to each segment. This option is preferred if the draft assembly quality is relatively low (e.g., a low coverage sequencing scheme).

The first option takes *O*(*K*) time, where *K* is the number of validated segments. The second option can take much longer time, because it requires to call BLASR or Nucmer for another sequence alignment. This new alignment is then used for consensus of the validated segments.

### Reassembling

In this step, our goal is to reassemble the extracted segments (e.g., Fig. 1 c is a possible reassembly). The available information for closing induced gaps hopefully is within the remaining unaligned long reads. Therefore, we next utilize these remaining long reads using a graph-based theoretical model — which also appeared as the scaffolding problem proposed by Igor et al. [14].

Prior work on this scaffolding problem was to reduce it to the vertex disjoint problem that is *NP*-hard, but no complexity analysis was given for the scaffolding problem. Here, we show that this scaffolding problem is indeed *NP*-hard. We also prove that the algorithm presented in ScaffMatch [14] is a 2-approximation of an optimal scaffolding using our independent formulation of this model.

#### Graph model construction

**Fig. 3:**
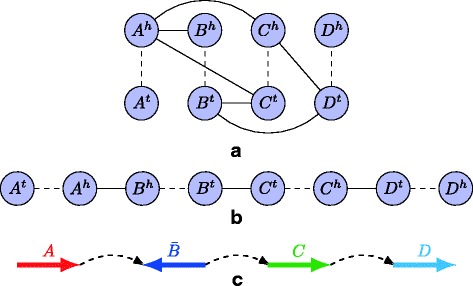
An example of our underlying graph model. **a** The graph constructed for four segments. Each dashed edge represents a segment from tail (5’-end) to head (3’-end). Each solid edge is for a bundle of long reads. **b** A possible ordering of the vertices/segments. This ordering will also decide the orientation of the segments in the resulting scaffolds. **c** The actual ordering and orientation based on the result of **b**

Our graph model (see Fig. 3 for an example) is derived from the concept of breakpoint graphs [24–26]. In a breakpoint graph, each gene is represented by two vertices, indicating the 5’-end and 3’-end of the gene. If two genes are consecutive in a scaffold, a colored edge will be added to connect the two corresponding vertices. Following the same idea, we view each validated segment as two vertices, and a dashed edge is added between these two vertices. A solid edge can be added between two vertices in the graph if there is a long read bridging them. Similar edges can be merged in this step. If there are still multiple edges between two vertices, we consider using the one with the largest support (the number of long reads) (see Fig. 3 a). This construction of the model has the advantage that in the successive steps of the algorithm, we do not have to consider the directions of the vertices (as shown in Fig. 3 b and c). Prior approaches of MAIA [13] and Medusa [27] both require the directions of vertices and edges to be determined. The directions are usually determined in two separate phases, which may result in accumulative assembly errors, given that both phases are *NP*-hard problems (and therefore approximated). This 2-phase difficulty was addressed by both our model and the similar graph model in ScaffMatch [14].

#### *NP*-hardness of the scaffolding problem

In this graph, an alternating path that starts at a dashed edge, followed by a solid edge, …, and ends at a dashed edge, is a possible scaffold (as shown in Fig. 3 b). Since each validated segment should ideally appear in exactly one scaffold in the final output assembly (i.e., no repeats, see Fig. 3 c), the objective of the problem is to find a set of (vertex-disjoint) alternating paths that covers all the dashed edges exactly once. In addition, we can weight the solid edges between vertices by the number of long reads that support that connection. Thus, the objective is to find a maximum weighted alternating path cover.

Formally, the scaffolding problem is defined as follows.

##### **Definition 1**

Given a weighted undirected graph *G*=(*U,U* ^′^;*E*), where *U*={*u*_1_,*u*_2_,…,*u*_*n*_}, *U* ^′^={*u*1′,*u*2′,…,*un*′}, and *E* is a set of dashed edges and solid edges. The dashed edges are exactly {(*u*_1_,*u*1′), (*u*_2_,*u*2′),…,(*u*_*n*_,*un*′)}. The solid edges may connect any two vertices in the graph. Each solid edge is assigned a positive edge weight, and the edge weight for each dashed edge is 0. An alternating path is defined as a simple path that starts at a dashed edge, followed by a solid edge, then a dashed edge, then a solid edge, …, and ends at a dashed edge. The objective is to find a set of alternating paths such that each vertex and each dashed edge appear in the paths exactly once (i.e., an alternating path cover), and the sum of the edge weights in all paths is maximized.

To prove that this problem is *NP*-hard, we prove that the decision version of the problem is *NP*-complete.

##### **Theorem 1**

Given a weighted undirected graph *G*=(*U,U* ^′^;*E*) defined as above, and a parameter *k*, the problem of determining whether there exists an alternating path cover whose edge weight sum is at least *k* is *NP*-complete.

##### *Proof*

Given any set of paths, we can easily verify whether they form an alternating path cover and the weight sum is at least *k* in polynomial time. Hence, the decision version of the scaffolding problem is in *NP*.

**Fig. 4:**
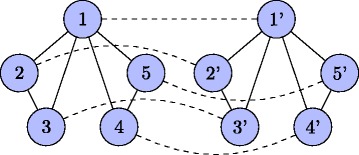
An example of the *NP*-completeness reduction. The reduction is from the Hamiltonian path problem to the decision version of the scaffolding problem. The instance of the Hamiltonian path problem is the 5-vertex graph on the left. The instance of the decision version of the scaffolding problem is the entire graph, including the dashed edges

To prove that the decision version is *NP*-complete, we reduce the Hamiltonian path problem (the undirected graph version) to this problem. Given an instance *I* of the Hamiltonian path problem *G*=(*V,E*), we make a copy of the vertex set *V* and *E* as *V* ^′^ and *E* ^′^. Let *E*∪*E* ^′^ be the solid edges in the instance *I* ^′^ of the decision version of the scaffolding problem. The dashed edges in *I* ^′^ are constructed by connecting the corresponding vertices in *V* and *V* ^′^. The edge weights of the solid edges are all 1’s and *k*=*n*−1. In this way, we construct an instance of the decision version of the scaffolding problem in polynomial time. Figure 4 shows an example of the construction.

(⇒) If there is a Hamiltonian path in *I*, we can denote the path as *u*_1_→*u*_2_→⋯→*u*_*n*_. Then we can construct a single alternating path *u*_1_→*u*1′→*u*2′→*u*_2_→*u*_3_→*u*3′→⋯. The path ends at *u*_*n*_ if *n* is even, and at *un*′ if *n* is odd. The edge weight sum of the alternating path is (*n*−1). Each dashed edge and each vertex in *I* ^′^ are covered exactly once, and edges (*u*1′,*u*2′), (*u*_2_,*u*_3_),… must be in the graph *I* ^′^ because the corresponding edges are in the Hamiltonian path, and thus in the original graph *I*.

(⇐) If there is an alternating path cover of edge weight sum at least (*n*−1) in the graph *I* ^′^, then the path cover must be a single alternating path. W.L.O.G, we can assume that the path starts at a vertex in *U*. Then the alternating path would be $u_{i_{1}} \rightarrow u_{i_{1}}' \rightarrow u_{i_{2}}' \rightarrow u_{i_{2}} \rightarrow u_{i_{3}} \rightarrow u_{i_{3}}' \cdots $. We can construct a Hamiltonian path $u_{i_{1}} \rightarrow u_{i_{2}} \rightarrow \cdots \rightarrow u_{i_{n}}$ since these edges must exist in the graph *I*.

Hence, the decision version of the scaffolding problem is *NP*-complete. □

#### The 2-approximation algorithm

For completeness, we also describe the algorithm presented in ScaffMatch [14]:

1. Find a maximum weighted matching by considering only the solid edges.
2. Add the dashed edges to the matching.
3. For each alternating cycle, change the smallest weight of its solid edges to -1, and run steps (1) and (2) until there is no cycle.

To prove that this is a 2-approximation algorithm, for simplicity, we relax step (3) such that we remove the smallest weight solid edge from each cycle. This relaxation is worse than the iterative algorithm above, but the worst case is equal. In fact, the current implementation of our algorithm follows this relaxed version. Our proof is similar to that for the ordinary path cover problem in [28].

##### **Theorem 2**

There is a 2-approximation algorithm for the scaffolding problem.

##### *Proof*

Let *M* ^∗^ be the weight sum of the maximum matching from step (1), *ALG* be the output value of the algorithm presented above, and *OPT* be the optimal value of the scaffolding problem. For each feasible solution, after removing the dashed edges, the solution must be a matching. Hence, we have *OPT*≤*M* ^∗^. On the other hand, for a cycle *c*_*i*_, let *k*_*i*_ be the number of solid edges in *c*_*i*_. And let *k*_*min*_= min{*k*_*i*_}. Since we remove the smallest weighted edge from each cycle. It follows that *ALG*≥*M* ^∗^·(*k*_*min*_−1)/*k*_*min*_. Thus *OPT*/*ALG*≤*k*_*min*_/(*k*_*min*_−1). In the worst case, *k*_*min*_=2. Hence, *OPT*/*ALG*≤2. □

##### **Remark 1**

If the output of the maximum weighted matching is improved (i.e., *k*_*min*_ is larger), then the approximation ratio of the algorithm will be better.

The time complexity of our 2-approximation algorithm is dominated by the maximum weighted matching. We applied the implementation version of the maximum weighted matching in the library LEMON [29] whose time complexity is *O*(*mn* log*n*), where *n* is the number of vertices and *m* is the number of edges in the graph for matching. Hence, the total time complexity for the 2-approximation algorithm is *O*(*Km* log*K*), where *m* is the number of edges, which in the worst case is the number of long reads, and *K* is the number of validated segments, which is proportional to the number of vertices in the graph.

## Abbreviations

SMS: Single molecule sequencing
